# Circulating tumor DNA as a prognostic marker in high-risk endometrial cancer

**DOI:** 10.1186/s12967-021-02722-8

**Published:** 2021-02-03

**Authors:** Weiwei Feng, Nan Jia, Haining Jiao, Jun Chen, Yan Chen, Yueru Zhang, Menghan Zhu, Chongying Zhu, Lifei Shen, Wenqing Long

**Affiliations:** 1 Department of Gynecology and Obstetrics, Ruijin Hospital, Shanghai Jiaotong University School of Medicine, 197 Ruijin 2nd Road, Huangpu District, Shanghai, 200025 People’s Republic of China; 2 Department of Gynecology, Obstetrics and Gynecology Hospital of Fudan University, Shanghai, 200091 People’s Republic of China; 3 Shanghai Gemple Biotech Co., Ltd., Shanghai, 201210 People’s Republic of China

**Keywords:** Circulating tumor DNA, High-risk, endometrial cancer, Recurrence

## Abstract

**Background:**

Currently, there is no reliable blood-based marker to track tumor recurrence in endometrial cancer (EC) patients. Liquid biopsies, specifically, circulating tumor DNA (ctDNA) analysis emerged as a way to monitor tumor metastasis. The objective of this study was to examine the feasibility of ctDNA in recurrence surveillance and prognostic evaluation of high-risk EC.

**Methods:**

Tumor tissues from nine high-risk EC patients were collected during primary surgery and tumor DNA was subjected to next generation sequencing to obtain the initial mutation spectrum using a 78 cancer-associated gene panel. Baseline and serial post-operative plasma samples were collected and droplet digital PCR (ddPCR) assays for patient-specific mutations were developed to track the mutations in the ctDNA in serial plasma samples. Log-rank test was used to assess the association between detection of ctDNA before or after surgery and disease-free survival.

**Results:**

Somatic mutations were identified in all of the cases. The most frequent mutated genes were *PTEN*, *FAT4*, *ARID1A*, *TP53*, *ZFHX3*, *ATM*, and *FBXW7*. For each patient, personalized ddPCR assays were designed for one-to-three high-frequent mutations. DdPCR analysis and tumor panel sequencing had a high level of agreement in the assessment of the mutant allele fractions in baseline tumor tissue DNA. CtDNA was detected in 67% (6 of 9) of baseline plasma samples, which was not predictive of disease-free survival (DFS). CtDNA was detected in serial post-operative plasma samples (ctDNA tracking) of 44% (4 of 9) of the patients, which predicted tumor relapse. The DFS was a median of 9 months (ctDNA detected) versus median DFS undefined (ctDNA not detected), with a hazard ratio of 17.43 (95% CI, 1.616–188.3). The sensitivity of post-operative ctDNA detection in estimating tumor relapse was 100% and specificity was 83.3%, which was superior to CA125 or HE4.

**Conclusions:**

Personalized ctDNA detection was effective and stable for high-risk EC. CtDNA tracking in post-operative plasma is valuable for predicting tumor recurrence.

## Background

Endometrial cancer (EC) is one of the most common invasive malignancies of the female genital tract. High-risk EC includes grade 3 endometroid EC (G3 EEC), serous carcinoma (SC), clear cell carcinoma (CCC), carcinosarcoma, and other rare types such as dedifferentiated carcinoma. Surgery is the standard treatment for EC, followed by chemotherapy, radiotherapy, or hormone (progesterone) therapy. High-risk ECs have much higher rates of metastasis and recurrence [1]; they account for only 20% of ECs but 48% of tumor-related mortality [2, 3]; thus, the prognosis of patients with high-risk EC remains poor after standard treatment [4].

Not all high-risk ECs relapse; the most commonly used tumor markers are CA125 and HE4, but they increase only when extrauterine metastasis exists and have relatively low sensitivity. Other markers include p53; microsatellite instability (MSI); POLE proofreading mutation; and hotspot mutations in *PIK3CA*, *KRAS*, *CDKNA2*, *CTNNB1*, *FBXW7*, *FGFR2*, *PPP2R1A*, and *PTEN* [5, 6]. However, tumor tissue biopsy is an invasive procedure and cannot reflect heterogeneity; moreover, consecutive monitoring cannot be achieved through one-time biopsy. Therefore, more sensitive, individually tailored, and easy-to-monitor markers to predict recurrence and prognosis are needed in order to provide individual treatments.

Circulating tumor DNA (ctDNA) can be detected in the plasma and serum of patients with advanced cancer [7], acting as a potential noninvasive means for characterizing the somatic genetic features of their tumors [8–12]. It can be used to monitor tumor recurrence and metastasis [13–17], evaluate prognosis [18–21], and to evaluate therapy responses [22, 23] through genetic and epigenetic tests. Combined with the assessment of circulating proteins, detecting mutations in ctDNA increased the specificity of nonmetastatic cancer detection to more than 99%, and it can also localize the cancer to a small number of anatomic sites in 83% of the patients among ovary, breast, and other six kinds of cancers [24]. This kind of “liquid biopsy” is simpler and more accessible than tissue biopsy and does not compromise tumor heterogeneity and successive monitoring.

Here, we analyzed the individual tumor genomes of high-risk EC and evaluated the sensitivity and specificity of this approach in long-term and dynamic follow-up. Our study aims to investigate the feasibility of the use of ctDNA combined with patients’ clinical characteristics in recurrence surveillance and prognostic evaluation of high-risk EC and provides a wealth of information on the potential utility as well as the limitations of ctDNA measurements for the assessment of patients with high-risk endometrial cancers.

## Methods

### Study design

The patients with high-risk ECs were recruited from Ruijin Hospital, Shanghai Jiaotong University, and Obstetrics and Gynecology Hospital of Fudan University and were treated with standard therapy. Tumor DNA was extracted from the pretreatment tumor biopsy during primary surgery and sequenced to obtain the initial mutation spectrum using a gene panel containing 78 cancer-associated genes. Serial plasma samples were collected from patients to assess the potential of ctDNA assays to predict relapse after treatment. Droplet Digital PCR (ddPCR) assays specific to patient-specific mutations were developed to track the mutation on ctDNA at baseline and in sequential plasma samples taken after surgery. The association between the detection of ctDNA before and after surgery and DFS was assessed.

### Patient cohort and sample collection

**Fig. 1:**
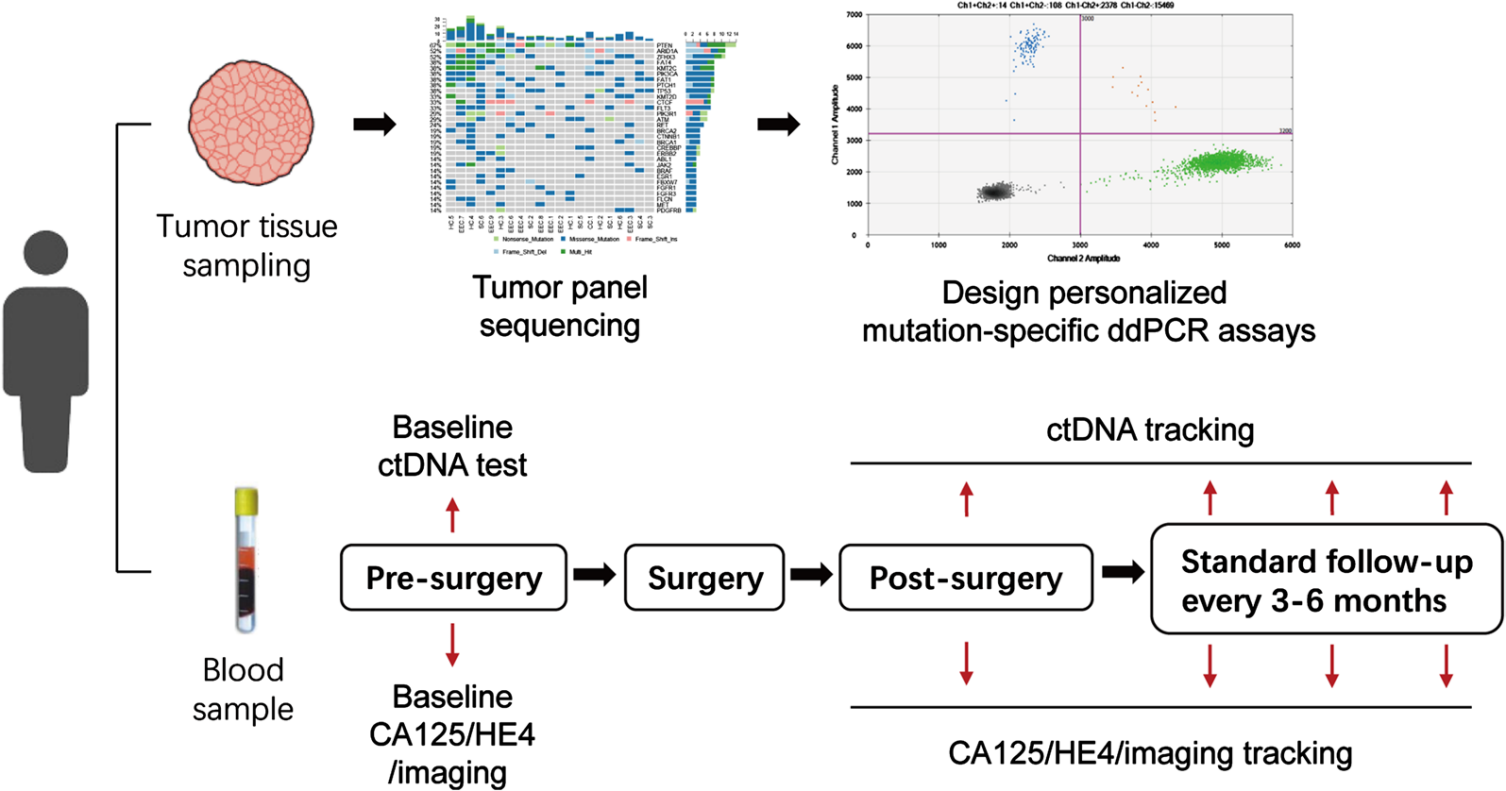
Personalized ddPCR assays for mutation tracking of ctDNA in plasma of patients with high-risk endometrial cancer. Tumor tissue sampling was performed during surgery and samples were subjected to TPS to identify somatic (tumor-specific) mutations. Personalized, patient-specific ddPCR assays were developed to detect the mutation in plasma DNA that was extracted from samples taken at baseline and during ctDNA tracking

After approval from the institutional review board of two hospitals, all patients provided written informed consent permitting the use of their tissue for research at the time of specimen collection. Patients with final pathological diagnosis of high-risk EC were recruited to the study, among which there were the following cases: 6 cases of serous carcinomas, 1 of endometroid carcinoma G3, 1 of clear cell carcinoma, and 1 of dedifferentiated carcinoma. Other clinicopathological characteristics were collected including age, FIGO stage, tumor size, depth of invasion, node status, lymphovascular space invasion (LVSI), involvement of lower uterine segment, adjuvant therapy and prognosis (Additional file 1: Table S1). All of the patients received standard surgical treatment followed by standard chemotherapy or combined radiotherapy and chemotherapy if needed. After completion of standard treatments, the patients were followed in a follow-up program. Plasma samples were collected into Cell-Free DNA Collection Tubes (Roche)at baseline (before surgery), 6 days after surgery, and every 3–6 months during follow-up or until relapse (all the post-operative samples including single post-surgical and serial samples during the follow-up were termed as “ctDNA tracking” samples; Fig. 1). Serum CA125 and HE4 of each patient were simultaneously assessed at the same time point as ctDNA samples collected.

### Processing and DNA extraction from tumor samples

All surgical tumor samples were subjected to H&E staining to confirm that the biopsy samples had identical pathology as the final diagnosis and to ensure that the percentage of tumor cells was above 80%. Tumor DNA was isolated using the Genome DNA Extraction Kit (UnigeneDx) as per the manufacturer’s instructions. Germline DNA was extracted from the buffy coat DNA using the Genomic DNA extraction kit (cell or tissue) (UnigeneDx) as per the manufacturer’s instructions.

### Tumor Panel sequencing (TPS) of baseline tumor samples

Frozen tumor tissues from surgical procedures were sent to a service provider for DNA sequencing (Shanghai Gemple Biotechnology Co. Ltd). Total DNA was isolated as previously mentioned. Nucleic acids were quantified and qualified before library construction using Qubit and Agilent 2100 chip assays. Briefly, DNA libraries were prepared with a gene panel targeting 78 known cancer-associated genes, which is named as Tumor Panel (Additional file 2: Table S2), using the KAPA Hyper Prep Kit and custom designed NimbleGen probes (Roche) under standard procedure. After performing the quality control steps, the libraries were sequenced on an Illumina HiSeq platform. An average of 1.2–1.5G PE150 raw data of DNA libraries were generated (average depth ~ 1000X). Raw data were subjected to a standard informatics pipeline for quality control, read alignment, and variant calling. Tertiary analysis was performed using an in-house software to annotate the variants from the vcf file and integrate information from multiple databases.

### Development of mutation-specific ddPCR assays

Mutations from tumor tissue sequencing were selected to develop ddPCR assays only if they met two criteria simultaneously: 1) occurred in more than two patients and present in the cosmic database, and 2) the mutation allele frequency was over 30%, indicating it might be a founder mutation rather than a subclonal mutation. Before assay development, we ensured the variants were not germline mutations using sanger sequencing of genomic DNA from the blood samples of patients.

DdPCR was performed on a QX200 ddPCR system (Bio-Rad) using TaqMan chemistry. For each tumor mutation, we developed a specific ddPCR assay. In brief, plasmids containing both wild-type and mutant sequences were constructed as quality controls. Primers and Taqman probe pairs were custom designed. Mutant-specific probes were usually FAM fluorescence labelled while wild-type-specific probes were usually VIC fluorescence labelled. Annealing temperature and cycling condition were optimized and LOD and assay sensitivity were determined using serially diluted plasmids. Data analysis was performed using QuantaSoft software following the manufacture ‘s instruction.

### DdPCR analysis of circulating free plasma DNA

The blood collected in Cell-Free DNA Collection Tubes was processed within 5 days of sample collection and was centrifuged at 1600 g for 10 min followed by 16,000 g for 10 min; the plasma was stored at -80 °C until DNA extraction. DNA was extracted from 2 mL of plasma using the MagMAX™ Cell‑Free DNA Isolation Kit(Applied Biosystems)according to the manufacturer’s instructions. The circulating free DNA was stored in DNA Elution of 50 uL. The concentrations of all the circulating DNA samples were assessed using qubit 3.0 (Thermo Scientific). Next, patient-specific ddPCR assays were performed to track the mutation on ctDNA, at baseline, and in sequential plasma samples taken after surgery. In brief, 900 nM probes and 250 nM primers were mixed with 2 × Droplet PCR Supermix (Bio-Rad Laboratories, Hercules, CA, USA), 5 μL (10 ng to 30 ng) of template DNA, and H_2_O to generate 20 μL for each reaction. The reaction mixture was placed into the sample well of an DG8 cartridge (Bio-Rad). Then, 70 μL of droplet-generation oil was loaded into the oil well, and droplets were formed in the droplet generator (BioRad). After processing, the droplets were transferred to a 96-well PCR plate (Eppendorf). The PCR amplification was carried out on C1000 TouchTM Thermal Cycler (Bio-Rad) with the following thermal profile: hold at 95 °C for 10 min, 40 cycles of 94 °C for 30 s and 58 °C for 1 min (ramp 2 °C/s), 1 cycle at 98 °C for 10 min, and ending at 4 °C. After amplification, the plate was loaded on the droplet reader (Bio-Rad) and the droplets from each well of the plate were read automatically. QuantaSoft software was used to count the PCR-positive (FAM channel) and PCR-negative (VIC channel) droplets to provide absolute quantification of target DNA. The quantification measurements of each target were expressed as the copies number per 1 µl of reaction. %mut was calculated from the generated Poisson concentrations as follows: %mut = [FAM]/[FAM + VIC]*100. A mutation was only considered to be present according to the sensitivity of the assay.

### Statistical analysis

The agreement between mutational frequency assessed by TPS and by mutation-specific ddPCR on baseline tumor was analyzed by Bland–Altman plot on MedCalc software (Fig. 3a). The association of baseline ctDNA level with clinicopathological factors was assessed using the Mann–Whitney *U* or the Kruskal–Wallis H test where appropriate (Table 1). The primary endpoint of the study was to assess DFS in patients with and without detection of ctDNA using univariable survival estimates calculated using the Kaplan–Meier method, and survival differences were estimated using the log-rank test (Fig. 4a, b). Mann–Whitney *U* test of two independent samples was used to test the association between ctDNA copies/mutant copies/mutant allele fraction of plasma at baseline and relapse/no relapse (Fig. 4c). Fisher’s exact test was used to test the association between ctDNA detection and clinicopathological variables (Additional file 5: Table S5) and between ctDNA/CA125/HE4/imaging and tumor relapse, and the kappa index was calculated, which is commonly used to assess inter-rater reliability, to evaluate concordance between relapse status and the abovementioned four test results (Fig. 5d). A kappa index of 0.61–0.80 indicates substantial concordance, and higher values indicate high concordance. The prediction of DFS using clinicopathological variables and ctDNA/CA125/HE4/imaging was estimated by univariable logistic regression analysis (Additional file 6: Table S6), and multivariable Cox regression analysis was used to test the independent prognostic value of post-operative ctDNA detection, adjusted for stage, tumor size, myometrial invasion, and nodal status. All statistical analyses were performed with SPSS 21.0 or GraphPad Prism 5. All P values were two-sided.

**Table 1:** Clinicopathological factors associated with baseline ctDNA level

	Median cfDNA level copies per ml (median,interquartile range)	P value	Mutant copies per ml (median,interquartile range)	P value	Mutant allele fraction %	P value	ctDNA detection	P value
Age								
Below 60	184,370 (105,485–264,285)	0.796	370 (185–745)	1	0.2	0.793	2/3 (67%)	1
Above 60	56,330 (43,245–70,015)		60 (15–1395)		0.115		4/6 (67%)	
Pathology subtype								
Endometroid	64,060 (/)	0.42	60 (/)	0.467	0.09	0.568	1/1 (100%)	0.446
Clear cell	693,400 (/)		1840 (/)		2.65		1/1 (100%)	
Serous	45,030 (36,615–150,427.5)		215(15–932.5)		0.17		4/6 (67%)	
Dedifferentiated	72,000 (/)		0 (/)		0		0/1 (0%)	
FIGO stage								
I–II	38,230 (32,900–49,095)	0.05	0 (0–15)	0.017	0.07	0.025	1/4 (25%)	0.025
III–IV	184,370 (64,060–344,200)		1120 (370–1840)		2.65		5/5 (100%)	
Tumor size								
< = 5 cm	52,760 (37,745–94,137.5)	0.327	60 (45–137.5)	0.453	0.115	0.455	3/4 (75%)	0.655
> 5 cm	72,000 (48,600–344,200)		1120 (0–1840)		2.65		3/5 (60%)	
Depth of invasion								
Superficial	38,230 (32,900–77,187.5)	0.086	30 (0–137.5)	0.169	0.07	0.213	2/4 (50%)	0.371
Deep	72,000 (64,060–344,200)		1120 (370–1840)		2.65		4/5 (80%)	
Node positive								
Yes	184,370 (64,060–344,200)	0.05	1120 (370–1840)	0.017	2.65	0.025	5/5 (100%)	0.025
No	38,230 (32,900–49,095)		0 (0–15)		0.07		1/4 (25%)	
LVSI								
Yes	128,185 (54,450–304,242.5)	0.142	745 (92.5–1660)	1	1.425	1	2/3 (67%)	0.655
No	41,460 (38,230–52,760)		60 (30–60)		0.09		4/6 (67%)	
Involvement of lower uterine segment								
Yes	68,030 (52,465–276,150)	0.439	590 (15–1660)	0.51	1.37	0.599	2/3 (67%)	1
No	41,460 (38,230–112,915)		60 (30–215)		0.14		4/6 (67%)	

P values for association between clinicopathological factors and baseline ctDNA level (median cfDNA levels, mutant copies, mutant allele fraction and ctDNA detection rate) were determined. Advanced FIGO stage and node metastasis were associated with high level of mutant copies and mutant allele fraction of ctDNA (FIGO stage: p = 0.017/0.025, node status: p = 0.017/0.025) and high detection rate of ctDNA (p = 0.025, both)

## Results

### Tumor Panel sequencing and establishment of ddPCR assays

**Fig. 2:**
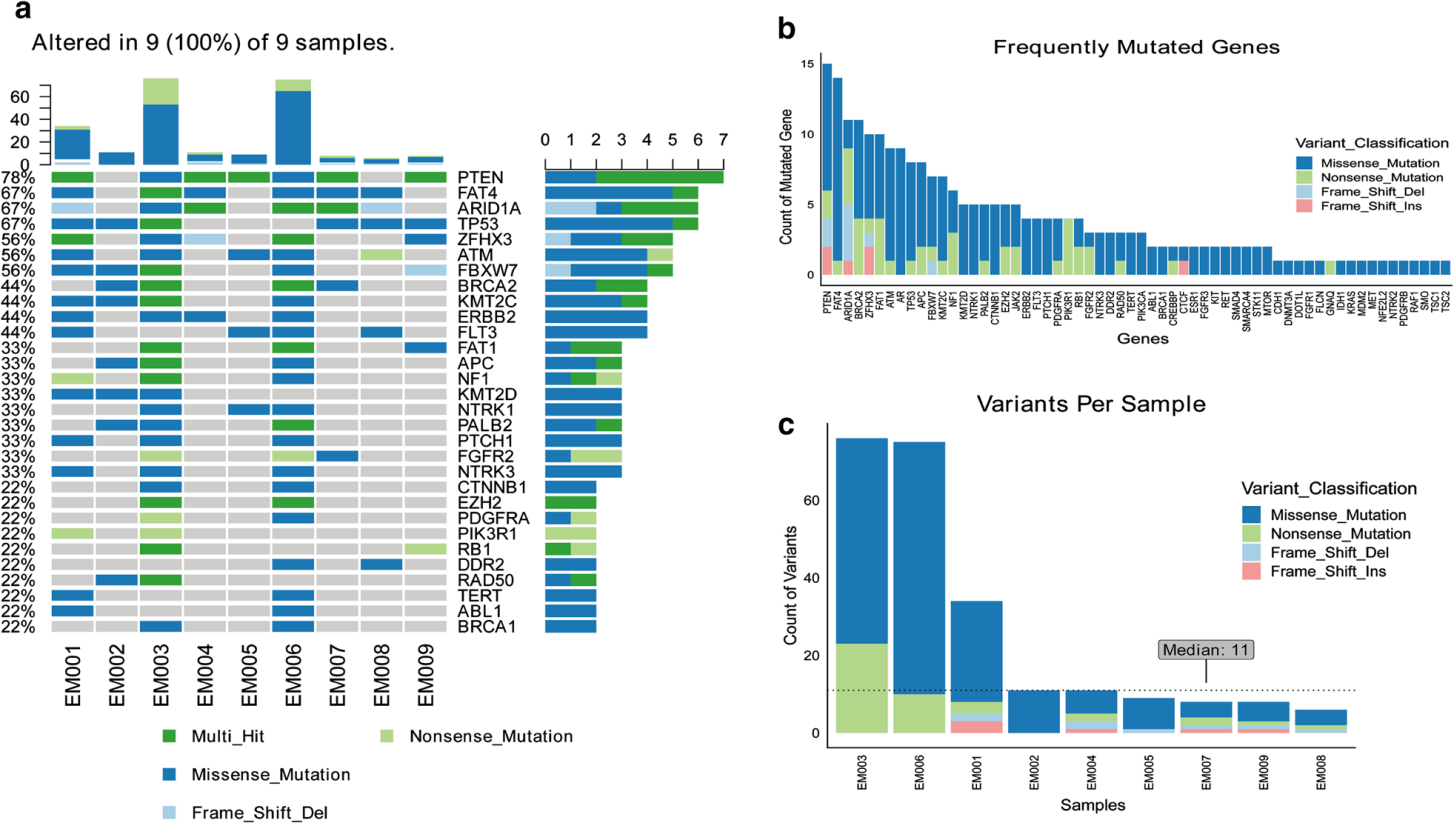
Tumor panel sequencing of nine samples. **a** The most frequently mutated genes (occurred in more than 50% of the cases) were PTEN, FAT4, ARID1A, TP53, ZFHX3, ATM, and FBXW7 (top 30 listed). **b** Rank of mutated gene count in all cases. **c** Variants per sample; most mutated variants were observed in EM003, EM006, and EM001; median count was 11. Most of the variants were missense mutations

To identify the disease-associated mutations, we first conducted NGS analysis of tumor samples. To this end, tumor samples and corresponding blood samples of all nine recruited patients were collected and tumor DNAs were analyzed using the Tumor Panel. Somatic mutations were identified in all of the cases. Somatic PTEN mutations were identified in 7 of 9 patients, which are of high frequency as previously reported [25]: 2 were missense mutations and 5 were multi-hit mutations (more than one type). *TP53* mutations were identified in all of the 6 serous carcinoma patients (EM001, 002, 003, 007, 008, 009), most of which were solely missense mutations, but these were absent in cases of non-serous carcinoma (EM004, 005, 006). In our analysis, the most frequently mutated genes were *PTEN*, *FAT4*, *ARID1A*, *TP53*, *ZFHX3*, *ATM*, and *FBXW7* (Fig. 2a). The results were partially in accordance with the mutation pattern of the subgroup of serous-like/copy number high of EC, which was characterized by mutations of *TP53, PIK3CA, FBXW7, PPP2R1A, PIK3R1, CHD4, PTEN,* and *CSMD3* (*PPP2R1A, CHD4*, and *CSMD3* were not included in Tumor Panel) [25]. We focused on *TP53, PTEN, PIK3CA, PIK3R1*, and *FBXW7*, and pathogenic or likely pathogenic mutations in these genes that were recorded in CLISING and cBioportal database (Additional file 3: Table S3) to determine whether they were related to recurrence or FIGO stage. However, there were no specific mutation patterns between recurrent/non-recurrent cases or advanced/early stage cases. Regarding the counts of mutated genes, *PTEN* was also the top one, and we could see multi-locus mutations in most of the genes (Fig. 2b). In EM001, 003, 006, the count of variants was much higher than that in other cases; however, it was not correlated with prognosis or any other clinical characteristics (Fig. 2c).

In order to track somatic mutations in plasma DNA and identify the presence of ctDNA, we chose one-to-three mutations of high frequency to design personalized ddPCR assays for each case (Additional file 4: Table S4), and germline mutations were ruled out. These mutations are usually reported frameshift, stopgain or nSNV variants on exons with high agreement on detection rate between TPS and ddPCR with certain ddPCR assay.

### Personalized tumor-specific ddPCR analysis was effective and reliable

After somatic mutations were identified for each case and personalized ddPCR assays were established, we investigated the potential utility of ctDNA analysis in high-risk EC in a prospectively cohort of 9 women presenting with high-risk EC (Fig. 2).

**Fig. 3:**
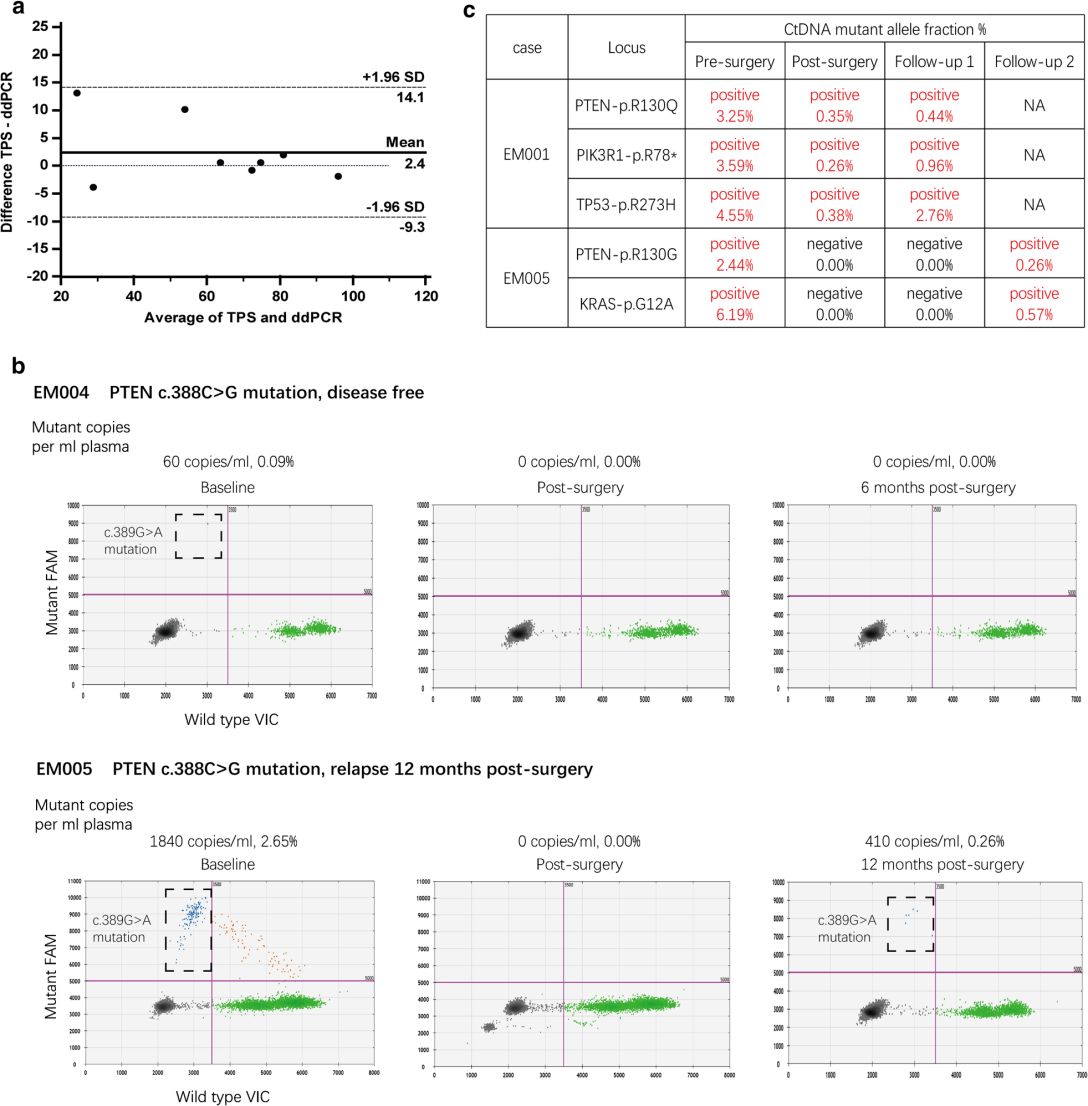
Personalized, mutation-specific ddPCR accurately quantifies ctDNA and was reproducible and reliable. **a** Bland–Altman plot of the agreement between mutational frequency assessed by TPS and by mutation-specific ddPCR on baseline tumor tissue DNA, with 95% CI of agreement (-9.3 and 14.1) indicated by dashed lines. Date points from eight samples are displayed and the different values of all samples are within 95% CI of agreement. **b** Tumors of EM004 and EM005 patients harbored the same somatic mutation (PTEN-c.389G > A). The patients had different outcomes with different ctDNA tracking results (complete time course is displayed in Fig. 4). In each ddPCR plot, green dots represent wild-type DNA (VIC-labeled), blue dots represent mutant DNA (FAM-labeled), brown dots represent droplets containing both wild-type and mutant DNA, and black dots represent droplets with no DNA incorporated. **c** Three/two different mutations were detected in patient EM001/EM005, and the ctDNA status remained the same among samples at each point in time

The data showed TPS and ddPCR analysis had a high level of agreement in the assessment of the mutant allele fractions in baseline tumor tissue DNA (Fig. 3a), demonstrating the robust ability to develop ddPCR assays for diverse mutations. DdPCR accurately quantified mutant DNA at single-molecule sensitivity, even in the presence of vast amounts of wild-type DNA (60 mutant copies in 64,060 cfDNA copies, 0.09%) (Fig. 3b). Tumors of EM004 and EM005 patients harbored the same somatic mutation (PTEN-c.389G > A), and ctDNA was not detected in the disease-free case while it was detected in the relapse case (Fig. 3b), demonstrating the effectivity and reliability of this method. In patients with more than one mutation identified in the primary tumor, we tracked all mutations in the plasma with stable agreement for present/absent mutation in the same plasma (Fig. 3c), emphasizing the reproducible and robust nature of the assays developed.

### Post-operative ctDNA status was correlated with disease-free survival

We determined whether ctDNA status analyzed by ddPCR was associated with tumor relapse. The personalized ddPCR assays were used to track mutations in serial plasma samples collected as previously mentioned. We assessed the correlation between relapse and ctDNA status at different time points. Consistent with previous observations [16], ctDNA was detected in 67% (6 of 9) of the baseline plasma samples. Baseline level including median cfDNA level, mutant copies, mutant allele fraction, and ctDNA detection rate were associated with the advanced status of the disease such as FIGO stage and node status, but not correlated to other clinicopathological characteristics including age, pathology subtypes, tumor size, myometrial invasion, LVSI, and involvement of lower uterine segment (Table 1).

**Fig. 4:**
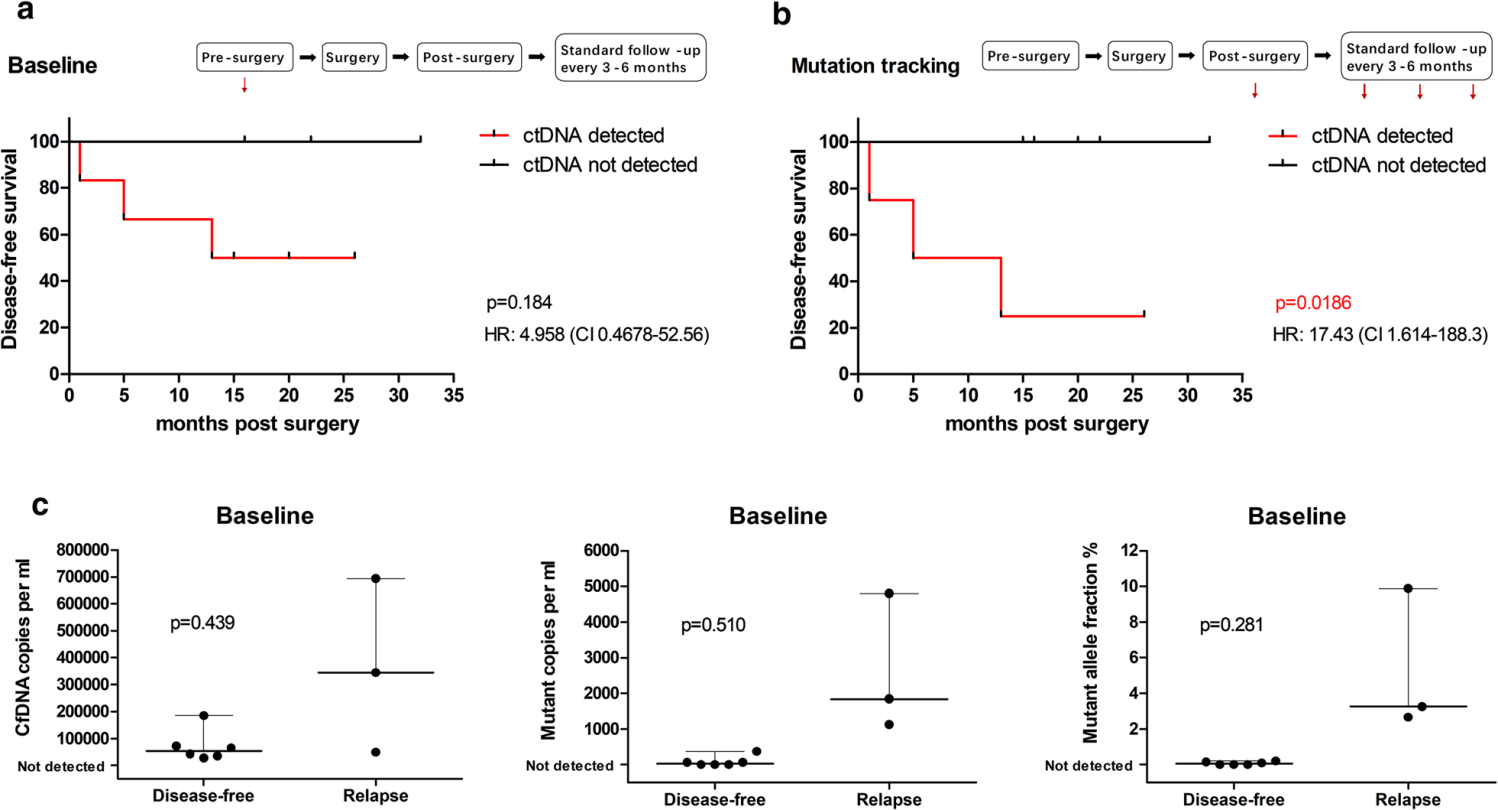
Correlation between tumor relapse and pre/post-operative ctDNA detection. **a** Tumor relapse was not predicted by analysis of baseline ctDNA. DFS according to the detection of ctDNA in the baseline plasma sample. P value determined by log-rank test (HR 4.958 [95% CI 0.4678 to 52.56]). **b** CtDNA tracking in post-operative plasma samples predicted tumor relapse. DFS according to the detection of ctDNA tracking samples. P value determined by log-rank test (HR 17.43 [95% CI 1.614 to 188.3]). **c** CfDNA copies, mutant copies, and mutant allele fraction of plasma at baseline in patients who relapsed/not relapse during follow-up. P value determined by Mann–Whitney U test. CtDNA associations with other clinicopathological characteristics are in Additional file 5: Table S5

CtDNA detection at baseline, i.e. before any treatment, was not predictive of DFS (Fig. 4a). CfDNA and ctDNA levels at baseline were higher in patients who relapsed than in those who did not relapse (cfDNA copies: median of 344,200 versus 52,760/mL; mutant copies: median of 1840 versus 30/mL; mutant allele fraction: median of 3.25% versus 0.045%, for relapse and DFS, respectively), although not at a statistically significant level (Fig. 4c).

CtDNA tracking in serial post-operative plasma samples predicted tumor relapse. We then assessed the potential of ctDNA tracking to report tumor relapse. CtDNA was detected in post-operative blood tests in 44% (4 of 9) of the patients (Fig. 4b), with highly variable mutational loads (median of 900 copies/mL; range, 290 to 81,300 copies/mL, mutant allele fraction from 0.15% to 79.47%). In these samples, ctDNA detection was predictive of tumor relapse (DFS: median of 9 months [ctDNA detected] versus median undefined [ctDNA not detected]; hazard ratio [HR], 17.43 [95% CI, 1.614 to 188.3]) (Fig. 4b). In ctDNA tracking, in accordance with baseline values, ctDNA detection was correlated with FIGO stage and node metastasis (Additional file 5: Table S5), which along with tumor size and myometrial invasion, were predictors of tumor relapse (Additional file 6: Table S6). These results suggest that post-operative ctDNA detection is closely related to tumor relapse, although due to small sample size, we cannot statistically prove that ctDNA is an independent prognostic factor in a multivariate cox regression analysis (P = 0.336, HR, 0.007, 95% CI 0.000–164.011).

### CtDNA was superior to CA125 or HE4 in detection of tumor relapse

**Fig. 5:**
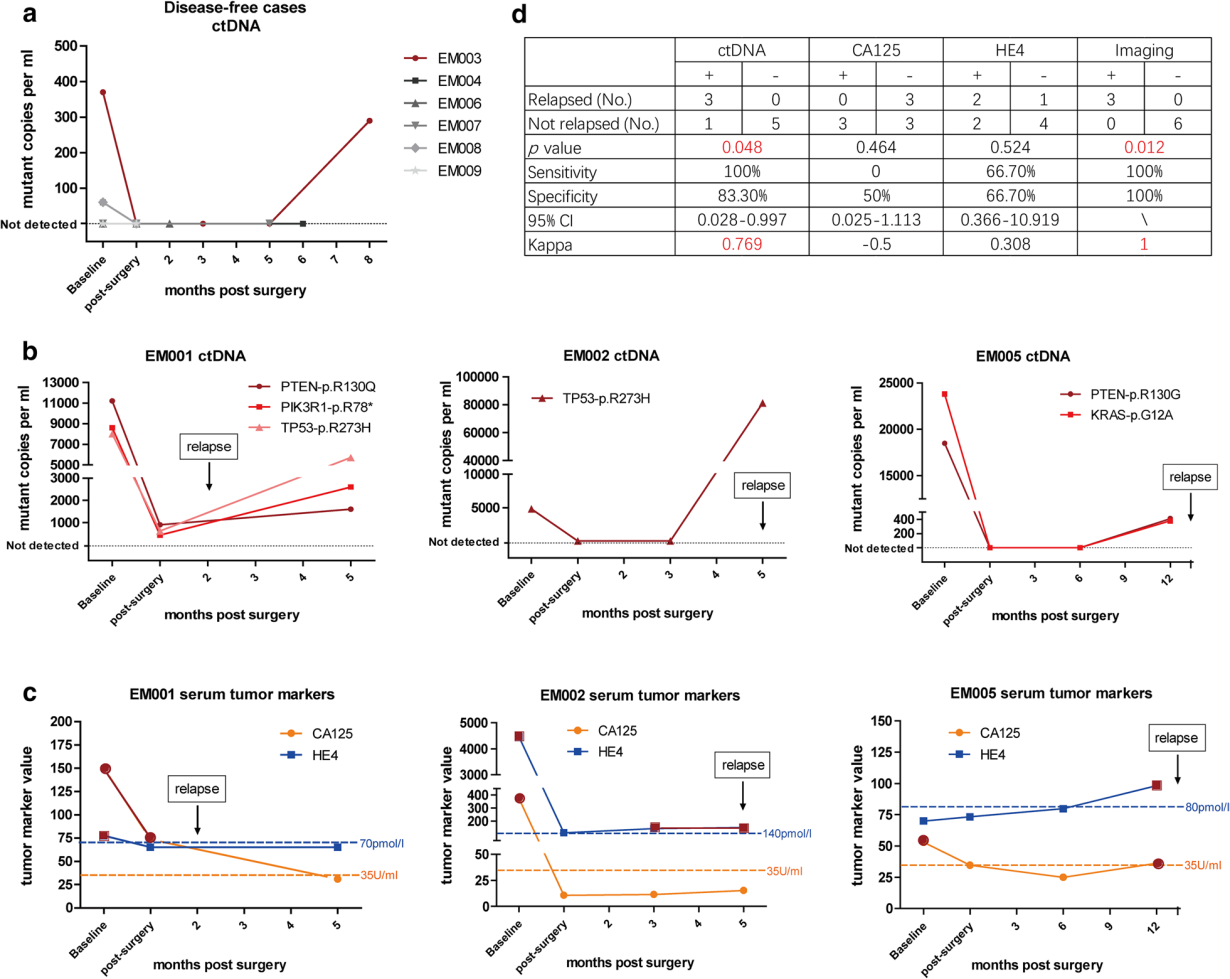
CtDNA and CA125/HE4 detection in disease-free and relapsed patients. **a** CtDNA tracking profile of 6 patients who are currently disease free at baseline and after standard treatment of high-risk endometrial cancer. Mutations were undetectable in the post-surgical follow-up periods in 5 of 6 patients. The remaining patient (EM003, red), with stage IIIC2 serous carcinoma showed ctDNA at 8 months after surgery, but did not have clinical relapse at the time of reporting (26 months after surgery). **b** CtDNA tracking profiles from three patients who experienced relapse at baseline and after standard treatment. CtDNA was detected in all cases at the time of or before relapse. **c** CA125 and HE4 tracking profiles from three patients who experienced relapse at baseline and after standard treatment. Orange dash lines denote the upper normal limit of CA125 in our hospital (35U/mL), blue dash lines denote different upper normal limits of HE4 with different age ranges (EM001, 50–59 years old, 70 pmol/L; EM002, above 70 years old, 140 pmol/L; EM005, 60–69 years old, 80 pmol/L). **d** Table showing ctDNA, CA125, HE4, imaging, and tumor relapse. CtDNA had higher sensitivity and specificity than CA125 and HE4 and had comparable performance with imaging in tumor relapse detection

Commonly used epithelial tumor markers CA125 and HE4 were concurrently monitored whenever ctDNA was analyzed. Of the patients who did not relapse, 5/6 did not show ctDNA in any post-operative plasma sample (P = 0.048). One patient (EM003) had ctDNA detected 8 months after surgery but stayed disease free until 26 months after surgery in the follow-up period (Fig. 5a). Of the patients who relapsed in the follow-up period, all cases (3/3) showed ctDNA in post-operative plasma samples (Fig. 5b).

The performance of common tumor markers was less reliable than ctDNA detection. CA125 was negative in all relapsed cases with an extremely high false-negative rate, and half of the disease-free cases had CA125 positive (3/6). HE4 had both sensitivity and specificity of 66.7% in estimating tumor relapse, with a kappa index of 0.308, which only suggested a fair concordance of this marker and tumor relapse estimation (Fig. 5c). Imaging had a perfect consistency with relapse, with 100% sensitivity and specificity. In general, the sensitivity of post-operative ctDNA detection to estimate tumor relapse was 100%, and the specificity was 83.3%, with a kappa index of 0.769, which indicated substantial concordance of post-operative ctDNA detection and tumor relapse (Fig. 5d).

## Discussion

Our study focus on high-risk ECs of higher malignancy (G3 EEC, SC, CCC, carcinosarcoma, etc.) since most of the endometroid ECs are of relatively lower risks of recurrence after standard treatments and ctDNA monitoring is not of great value. We explored the value of personalized ctDNA detection in recurrence monitoring and prognosis evaluation in high-risk EC. Moreover, we did complete sequential ctDNA monitoring during the follow up as well as the comparison with traditional serum biomarkers, which is not included in most of other studies. We found that the methods were effective and stable; tumor recurrence was correlated with the status of ctDNA tracking samples and ctDNA was a better biomarker than conventional serum tumor marker CA125 or HE4.

Some studies found that more than a half of cfDNA mutations in patients with cancer might come from clonal hematopoiesis [26], so cfDNA mutations may not represent tumor mutations. However, mutations designed in ddPCR assays came from tumor mutations, and we ruled out germline mutations by testing for matched white blood cells before plasma ctDNA detection. Moreover, our results showed high agreement on mutant allele fractions between NGS and ddPCR on the same mutational site (Fig. 3a); the same mutation in different samples varied according to different prognosis (Fig. 3b), and identical interpretations were observed on different sites in the same sample (Fig. 3c). These results suggest the rigor of the method used in this study.

A large panel of 78 cancer-associated genes was used in our study including all the known molecular therapeutic targets. Some of the studies used similar panels as ours [27, 28] while others used small panels containing hotspot mutation sites of 2–4 genes [29, 30]. Large panels contain more variation, making a higher detection rate of mutations, however, the cost of sequencing and ddPCR assay both rise due to dispersed mutational sites. Small panels may miss some mutations but are more suitable for clinical application with lower costs.

The detection rate of pre-surgical ctDNA in our study was related to FIGO stage and node positivity, which is similar with other studies, suggesting that it is closely related to tumor load. In our study, all the three ctDNA negative cases were FIGO stage I, and ctDNA was detected in only 1/4 of stage I to II cases but 5/5 stage III to IV cases. It is similar with the results that ctDNA was detected in 18% of early stage cases [30]. Thus, this method is more significant in advanced cases for evaluating the effect of tumor reduction and recurrence monitoring.

The detection rate of pre-surgical ctDNA is seemingly related to pathological types too. We reported the highest detection rare of ctDNA at surgery (67%) compared with other studies, probably because only high-risk pathologic types of ECs were included. A detection rate of 41.2% was reported [27] when the proportion of high-risk ECs was 28.3%, and 33% was reported [30] when only endometroid EC was included. It is reasonable since with higher degree of malignancy, the tumor is more likely to spread and be detected.

Apart from the pre-surgical (base line) ctDNA measurement, we did complete sequential ctDNA monitoring during the follow up (plasma samples collected on the sixth day after surgery and at three-months interval thereafter). We observed the clearance of ctDNA in most of the cases on the sixth day after surgery, indicating that the half-life of ctDNA is very short, so it is very sensitive since it responds quickly to the change in tumor load. Post-operative ctDNA ((Fig. 4b) but not baseline ctDNA (Fig. 4a) predicted tumor relapse. The data showed the difference in cfDNA quantity, mutant copies, or mutant allele fractions between relapse and non-relapsed groups, but there was no statistical significance maybe due to the small sample size. The abundance of cfDNA, mutant copies, or mutant allele fractions at baseline was correlated to FIGO stage and node metastasis, indicating that patients with advanced disease tend to have higher quantity of cfDNA due to higher tumor load. The recurrence rate was higher in advanced disease, and pre-surgical ctDNA may relate to recurrence through tumor stage, so it is not as specific as ctDNA of post-operative serial samples in predicting recurrence, since the latter performs better in evaluating residual tumor load.

CA125 and HE4 are conventional tumor markers for EC. Our results showed ctDNA was better than serum tumor markers CA125 and HE4 in recurrence evaluation (Fig. 5c, d). However, ctDNA was not detected earlier than imaging findings in relapse cases, probably because ctDNA detection and imaging were carried out at the same time during follow up, and early detection might have been missed due to the relatively long detection intervals. Previous studies showed the detection of ctDNA had a median of 7.9 months (range, 0.03 to 13.6 months) lead time over clinical relapse [17], suggesting the potential of ctDNA in early prediction of tumor relapse.

There were also some limitations to this study. CtDNA was not detected in all the cases. Most cases (3/4) in the early stage (FIGO stage I to II) remained negative and all cases (5/5) in the advanced stage (FIGO stage III to IV) were positive for baseline ctDNA, suggesting that ctDNA was not specific enough in cases of confined lesions but more suitable in cases of high tumor load. The EM003 case was positive at nine months after surgery, but the patient remained disease free for 2 years since then. Therefore, enough input should be used and the threshold value of ddPCR analysis should be set carefully to reduce the false-positive rate. A small sample size, short follow-up time, and relatively long follow-up interval may have caused bias in this study. The genes included in NGS panel are not custom-made for EC, so some of the key genes related to EC may have been missed and the design of ddPCR assay may have been restricted. The high cost of NGS and ddPCR assay also limits the clinical utility of personalized liquid biopsy.

## Conclusions

CtDNA was valuable in monitoring high-risk EC relapse during post-operative follow-up as a prognostic marker, and it had better performance than traditional serum tumor markers. Liquid biopsy should evaluate drug sensitivity in relapse cases when biopsy samples cannot be assessed. CtDNA detection used as recurrence or drug response monitoring for high-risk ECs needs to be further explored and the costs need to be decreased in future.

## Supplementary Information

**Additional file 1: Table S1.** Clinicopathological characteristic of detected cases.

**Additional file 2: Table S2.** Genes included in Tumor Panel.

**Additional file 3: Table S3.** Pathogenic/Likely pathogenic mutations* in detected cases.

**Additional file 4: Table S4.** DdPCR assays for detected cases.

**Additional file 5: Table S5.** Relationship between ctDNA tracking and clinicopathological characteristics.

**Additional file 6: Table S6.** Predictors of disease-free survival.

## Data Availability

All the data were presented in the article as tables and figures and supplemental tables. The original tumor panel sequencing data were available as request to corresponding author.

## Abbreviations

CtDNA: Circulating tumor DNA
EC: Endometrial cancer
DdPCR: Droplet digital PCR
DFS: Disease free survival
G3EEC: Grade 3 endometroid endometrial carcinoma
SC: Serous carcinoma
CCC: Clear cell carcinoma
MSI: Microsatellite instability
TPS: Tumor Panel sequencing
